# From efficacy to effectiveness: child and adolescent eating disorder treatments in the real world (part 1)—treatment course and outcomes

**DOI:** 10.1186/s40337-022-00553-6

**Published:** 2022-02-21

**Authors:** Mima Simic, Catherine S. Stewart, Anna Konstantellou, John Hodsoll, Ivan Eisler, Julian Baudinet

**Affiliations:** 1 Maudsley Centre for Child and Adolescent Eating Disorders (MCCAED), Maudsley Hospital, De Crespigny Park, Denmark Hill, London, SE5 8AZ UK; 2 Institute of Psychiatry, Psychology and Neuroscience, King’s College London, 16 De Crespigny Park, Denmark Hill, London, SE5 8AF UK

**Keywords:** Child, Adolescent, Anorexia nervosa, Bulimia nervosa, Family therapy for anorexia nervosa, Family therapy for bulimia nervosa, Family based treatment

## Abstract

**Background:**

Findings from randomised control trials inform the development of evidence-based eating disorder (ED) practice guidelines internationally. Only recently are data beginning to emerge regarding how these treatments perform outside of research settings. This study aimed to evaluate treatment pathways and outcomes for a specialist child and adolescent ED service across a five-year period.

**Methods:**

All consecutive referrals between August 2009 and January 2014 seen at the Maudsley Centre for Child and Adolescent Eating Disorders in London were included. Data are reported on for all young people who were offered treatment (N = 357).

**Results:**

Most young people referred to the service were diagnosed with anorexia nervosa (AN)/Atypical AN (81%). Treatment for AN/Atypical AN (median 11 months) was predominantly ED focused family therapy (99%). Treatment for bulimia nervosa (BN)/Atypical BN (median seven months) was most commonly a combination of cognitive behavioural therapy and ED focused family therapy (87%). At discharge, 77% of the AN/Atypical AN group had a good or intermediate outcome and 59% of the BN/Atypical BN group reported no or fewer than weekly bulimic episodes. 27% of the AN/Atypical AN group had enhanced treatment with either day- and/or inpatient admissions (AIM group). The %mBMI at 3 months of treatment was strongest predictor of the need for treatment enhancement and more modestly EDE-Q and age at assessment. The AIM group at assessment had significantly lower weight, and higher ED and comorbid symptomatology and went on to have significantly longer treatment (16 vs. 10 months). At discharge, this group had significantly fewer good and more poor outcomes on the Morgan Russell criteria, but similar outcomes regarding ED and comorbid symptoms and quality of life. When analysis was adjusted for %mBMI at assessment, 1 and 3 months of treatment, differences in Morgan Russell outcomes and %mBMI were small and compatible with no difference in outcome by treatment group.

**Conclusions:**

This study shows that outcomes in routine clinical practice in a specialist community-based service compare well to those reported in research trials. The finding from research trials that early weight gain is associated with improved outcomes was also replicated in this study. Enhancing outpatient treatment with day treatment and/or inpatient care is associated with favourable outcome for most of the young people, although a longer duration of treatment is required.

**Supplementary Information:**

The online version contains supplementary material available at 10.1186/s40337-022-00553-6.

## Plain English summary

Most research reports on outcomes for clinical trials. This study aimed to evaluate outcomes in a ‘real world’ setting of a specialist child and adolescent eating disorder service (ED) in the UK. Case notes of 357 young people seen for treatment between August 2009 and January 2014 were reviewed. Demographic and treatment characteristics, physical health, ED symptoms, other psychological symptoms and quality of life data are reported. Most young people referred had anorexia nervosa or related difficulties and most received ED focused family therapy. At the end of treatment, the majority had a good or intermediate outcome, regardless of ED diagnosis. In a quarter of the young people, their treatment was enhanced with day or inpatient admissions. This group had more severe difficulties at assessment and had longer treatment but had similar outcomes at the end of treatment.

**Supplementary Information:**

The online version contains supplementary material available at 10.1186/s40337-022-00553-6.

## Background

Findings from randomised control trials (RCT) have informed the development of evidence-based practice (EBP) guidelines for treatment of eating disorders (EDs) across the world. A recent comparison of current EBP clinical guidelines [1] for EDs that included national guidelines from Australia and New Zealand [2], Germany [3], The Netherlands [4], Spain [5], the United Kingdom (UK) [6], the United States (USA) [7], Denmark [8, 9] and France [10] found notable commonalities and differences. All guidelines provide information on the treatment of anorexia nervosa (AN) and seven provide guidance on treatment setting.

Outpatient treatment is consistently recommended as the first-line therapy setting for all ED patients. Psychotherapy is considered a central part of treatment by all guidelines. Seven recommend family-based therapy (FBT) or family therapy focused on anorexia nervosa (FT-AN) as a first line treatment for AN in young people [2, 4–8, 10]. FBT/FT has also been recommended for young people with bulimia nervosa (BN) in four national guidelines [4, 6, 7, 9]. The German guideline recommend cognitive-behavioural therapy for young people with BN, but also emphasize the importance of including the young person’s family into treatment.

In the field of EDs, data is now emerging regarding how evidence-based treatments perform in everyday clinical practice. For adolescents with AN, results from naturalistic studies in private and public healthcare settings indicate outcomes are similar to those reported in RCTs [11–14]. Approximately 44–57% of young people reach weight restoration (when this is defined as achieving 95% mBMI) at one year from the start of FBT [11–14]. Furthermore, consistent with findings from RCTs, FBT in the community may be superior to other treatments at achieving weight restoration for adolescent AN [14]. Very little is known about bulimia nervosa and whether treatment outcomes in real world clinical settings matches RCTs findings.

When RCT and real-world specialist clinical setting outcomes are directly compared, the pattern of findings is similar. Irrespective of the treatment setting (RCT or specialist clinical practice), adolescents with AN with a higher weight at assessment are more likely to reach weight restoration by the end of treatment and they achieve it faster [12]. Nevertheless, there is some evidence that low weight young people (less than 81%mBMI) may achieve weight restoration more quickly in the RCT context, compared to non-research specialist clinical practice [11]. No direct comparison has been made of adolescent BN treatment outcomes across settings.

Despite promising outcomes for FBT/FT-AN, a proportion of young people do not respond and continue to need additional or enhanced treatment, regardless of treatment setting. These include individual treatments, medication, and/or intensive/day/residential/inpatient treatment programmes. Emerging data on the predictors of poorer treatment response indicate that low weight and higher ED obsessionality at start of treatment, lack of early weight gain in treatment (for those underweight) and higher levels of expressed emotion and criticism within the family are associated with poorer outcomes [15].

In a naturalistic cohort study of 3997 adolescents (aged 13–19 at initial entry) identified through the Swedish national quality register for EDs, treatment outcomes were analysed for inpatient, day patient and/or outpatient settings [16]. Approximately 60% were considered to have a low weight when entering treatment and almost as many had an AN diagnosis. At the end of treatment 55% of the participants were in remission and approximately 85% were within a healthy weight range. The average treatment duration was approximately 15 months. Participants who ended treatment prematurely (28.8%) had a decreased chance of achieving remission. Furthermore, patients who received family-based treatment and/or inpatient care were most likely to achieve remission at 1-year-follow-up [17].

Given the range and complexity of presentations to real world specialist services, clinical practice often includes various combinations of treatment modalities and settings, varied duration of treatment and a more flexible approach than is seen in research trials. This is to ensure all young people, including those with poorer treatment response, are provided best available care. However, what remains relatively understudied and less understood, is what predicts the need for more intensive treatment within the real-world clinical context and how effective these modifications/enhancements are. As such, clinicians can feel unsure when and how to enhance treatment within their day-to-day practice.

The primary aim of this study is to evaluate outcomes following treatment in a comprehensive specialist child and adolescent ED service, including predictors of enhanced treatment need and their outcomes. This will begin to build evidence for when and how to enhance treatment outside of controlled trials and what factors may predict the need for enhanced treatment. Specifically, this study has five main aims:

1. To describe the sample and characterise the treatment(s) provided by the service.
2. To evaluate the effectiveness of the treatment programme offered by the service.
3. To evaluate the extent to which outpatient treatment requires enhancement through day- and in-patient care and compare duration of treatment for the group of patients having additional treatments to the group of patients who had outpatient treatment on its own.
4. To analyse factors predictive of the need for treatment enhancement.
5. To compare outcomes of treatment for young people who required treatment enhancement compared to young people who had just outpatient treatment.

Seven-year follow-up data for the same cohort are presented separately [18].

## Method

### Setting

The Maudsley Centre for Child and Adolescent Eating Disorders (MCCAED) is a National and Specialist child and adolescent mental health service within the UK National Health System (NHS). MCCAED is the primary provider of specialist treatments for children and adolescents with EDs for an area of South East London with a population of approximately 2.2 million people.

The service provides a comprehensive evidence based treatment programme which includes psychiatric management, dietetics and physical health review, as well as the following psychological therapies for EDs: family therapy for AN (FT-AN), cognitive behavioural therapy (CBT) for BN, CBT for comorbidities (anxiety, depression, obsessive–compulsive disorder), family therapy for BN (FT-BN), multi-family therapy for AN (MFT-AN) and BN (MFT-BN), an intensive day treatment programme (ITP) for AN/Atypical AN and brief paediatric admissions for medically unstable patients.^1^ ITP was added to the service delivery in September 2010 [20]. Patients are only admitted to a general psychiatric adolescent unit or a specialist adolescent ED unit when they do not respond to out or day patient treatments or where physical or psychiatric risk precludes the initiation of these.

FT-AN includes some individual systemic work with the young person [21]. Analysis below considers young people to have received individual treatment if seen individually for four or more planned consecutive sessions during treatment.

Treatment within MCCAED is of variable length based on clinical need. Young people are discharged either to primary care if no further mental health input is needed, to community child and adolescent mental health services (CAMHS) for further psychiatric care for non-ED related difficulties, or to adult ED services if they turn 18 and continue to require specialist ED care.

### Procedure

A case note review was conducted to explore the treatment course and outcomes for consecutive referrals made to MCCAED from the local catchment area in South East London (approximate population 2,200,000) between 01/08/2009 and 31/01/2014. This service effectiveness evaluation was approved by the South London and Maudsley NHS Foundation Trust Child and Adolescent Mental Health service evaluation and audit committee.

### Patient sample

The sample consist of consecutive referrals to MCCAED who met inclusion criteria for the study:

1. International Classification of Diseases, 10th edition, (ICD-10) [22] diagnosis of anorexia nervosa, atypical anorexia nervosa, bulimia nervosa or atypical bulimia nervosa
2. Offered treatment at MCCAED
3. Referred from the MCCAED catchment area

Four hundred and eleven young people were referred to MCCAED from within the service catchment area during the study period. Of these, 10 did not attend assessment, six were referred to services more appropriate for their primary clinical needs and 32 did not meet the specific study ED diagnostic criteria at assessment. The remaining 357 were seen for treatment in MCCAED and their data are reported here.

Young people with a diagnosis of AN or Atypical AN were subsequently grouped into two groups on the basis of whether they received only outpatient treatment (OPT group) or whether their treatment was enhanced through day programme within MCCAED (for details of the programme see [19, 20] or inpatient admission to the specialist ED unit (Additional Intervention and Management—AIM group).

### Clinical and demographic data

All data were derived from routine clinical assessment. Diagnoses using ICD-10 criteria were made via clinical assessment compromising clinical interview with the young person and parent, medical examination and Eating Disorders Examination Questionnaire [23]. Demographic and clinical information and details of treatment pathways were extracted from clinical notes. This included age, gender, ethnicity, parents’ marital status, duration of illness prior to referral, interventions provided in MCCAED, admissions to in- and day-patient programmes, percentage median body mass index (%mBMI) and discharge or onward referrals made following treatment. The duration of illness prior to referral was estimated at clinical interview following parent and child description of symptom onset. Where information was unclear it was reviewed by a senior clinician and the treating therapist, and a consensus rating was agreed.

Weight data (%mBMI) were extracted for each individual at assessment (n = 290/290), one (n = 287/290), three (n = 268/274), six (n = 243/246), nine (n = 190/194), and twelve months (n = 146/147) into treatment and at discharge from the service (n = 287/290). The lower numbers of data points for six, nine and twelve months partly reflects that people were being increasingly discharged from treatment by this time. Menstrual status was extracted at assessment and discharge.

### Morgan Russell outcome

Morgan Russell Scales General Outcome Score [24] modified by Russell and colleagues was used to classify treatment outcomes (Good ≥ 85%mBMI with menstruation or premenarchal and no bulimic symptoms; Intermediate ≥ 85%mBMI without menstruation or bulimic symptoms averaging < 1 per week over the last month; Poor: below 85%mBMI or bulimic symptoms averaging ≥ 1 per week over the last month). Data pertaining to bulimic behaviours was derived from both EDE-Q reports and from assessment and discharge clinical records. Three cases were missing end of treatment weight data and could not be rated on the Morgan Russell criteria.

### Self-report measures

A standard battery of self-report questionnaires was sent for completion at home prior to attending the clinic for assessment. Data derived from the following questionnaires was included:

Eating Disorder Examination Questionnaire (EDE-Q) [23, 25]: The EDE-Q is a 28-item self-report questionnaire measuring ED behaviours and cognitions. It includes restraint, weight concern, shape concern and eating concern scales which are combined to provide a global scale. The EDE-Q is widely used and has good psychometric properties [26]. It has demonstrated good internal consistency in a recent sample of adolescents with EDs [20] and in the follow-up study of this sample.

Mood and Feelings Questionnaire (MFQ) [27, 28]: The MFQ is a 33-item self-report measure assessing symptoms of depressive disorder in young people. The total score was used here, with a score of 27 or higher [29] indicating the presence of a mood disorder. The MFQ been shown to have good validity, reliability and internal consistency with adolescents [30].

Screen for Child Anxiety Related Emotional Disorders (SCARED) [31]: The SCARED is a 41-item screening self-report measure based on DSM-IV symptom criteria for anxiety disorders. The total score was used here, with a cut off of 25 indicating the presence of an anxiety disorder. It has high internal consistency and discriminant validity [31].

Children’s Obsessional Compulsive Inventory (ChOCI) [32]. The ChOCI is a 38-item self-report measure of specific compulsions and obsessions and their severity. The ChOCI total impairment score of > 17 was used to define the cut-off point for caseness with reported sensitivity of 88% and a specificity of 95%. It has good validity and test–retest reliability [32].

Quality of Life (QoL). A single item, global quality of life question (rated 1–10), from the EDQLS (Eating Disorder Quality of Life Scale) [30] was used. This correlates well (0.73) with the complete measure [33].

### Statistical analyses

The Statistical Package for Social Sciences (SPSS) version 23.0 [34] and R 4.05 with the rms package [35] were used for all analyses.

*Analyses 1* to characterise the patients seen and treatments delivered. Descriptive statistics are reported for key variables that summarise the presentation of young people receiving treatment. Chi squared and Mann Whitney analyses were used to test differences in comorbidities and treatment length between AN/Atypical AN and BN/Atypical BN groups.

*Analyses 2* to evaluate the effectiveness of the treatment programme. A series of independent t tests tested changes in clinical symptoms of EDs and common comorbidities for those with AN/atypical AN. Descriptive statistics summarise outcomes according to Morgan Russell criteria and need for ongoing treatment in primary or secondary care.

*Analyses 3* To evaluate the extent to which outpatient treatment requires enhancement through day- and in-patient care. Descriptive statistics summarise frequencies of additional day and/or inpatient treatments. Length of time in treatment (time to discharge) was assessed using survival analysis. Kaplan Meier survival curves were constructed to show the risk of discharge (time-to-event) through treatment.

*Analyses 4* In this analysis logistic regression models were used to test whether baseline characteristics and %mBMI at one and three months predicted the need for additional treatment. Baseline characteristics included age, illness duration, baseline EDE-Q, SCARED, ChOCI, QoL. Percentage of median BMI (%mBMI) at assessment, one month and three months of treatment were also included. The association of these factors with additional treatment were reported as odds ratios with 95% CI (confidence intervals) to estimate uncertainty. See Additional file 1 for further details on Analysis 4.

*Analyses 5* To compare outcomes of OPT/AIM treatments, Chi squared analyses were used to test differences in Morgan Russell (MR) global outcomes and series of independent t tests were used to test differences for other outcomes between the two groups at discharge. As the AIM group was assigned to additional enhanced treatment at some point during treatment it was postulated that %mBMI at 1 and 3 months was on the causal pathway between assessment and outcomes and had direct effects on outcome. To assess equivalence for discharge outcome between OPT and AIM groups, MR global outcomes were fitted into an ordinal regression model with added day/inpatient treatment as a predictor but controlled for %mBMI at 1 and 3 months.

There were missing data, both in terms of outcomes (see tables in the results section) and in terms of predictors. For the set of variables for Analysis 4 and 5 (the need for additional treatment and time to discharge) the overall rate of missingness was 9.5%. Little’s Missing Completely at Random (MCAR) test was significant and so we assumed the data was Missing at Random (MAR). Hence, we used multiple imputation with predictive mean matching and 25 sets of imputation to allow for this whilst including all available data.

Young people who completed the questionnaires were not significantly different from those who did not, in regard to age, duration of untreated disorder prior to assessment, baseline scores on self-report measures at assessment, %mBMI at assessment and discharge from the service and length of treatment.

## Results

### Clinical and demographic characteristics of the young people referred to the service

Descriptive statistics of the socio-demographic and clinical data for all young people treated by MCCAED during the evaluation period are presented in Table 1. The great majority of young people self-identified as female (n = 332, 93%) and White British (n = 277, 78%). The majority had a diagnosis of AN or Atypical AN (n = 290, 81%). The rest had BN or Atypical BN (n = 67, 18.7%). A high percentage of all subjects reported symptoms of comorbid anxiety, depression symptoms and obsessive–compulsive symptoms that were above the clinical cut-off for caseness. Significantly more young people with BN/Atypical BN were over the cut off indicating the presence of a depressive disorder (χ^2^(1) = 5.27, *p* = 0.02) compared to young people with AN/Atypical AN.

**Table 1:** Baseline socio-demographic and clinical characteristics of whole sample (N = 357)

		n (%)	Mean (SD)
*Demographic data*			
Age (years)			14.75 (1.9)
Gender	Female	332 (93.0%)	
	Male	25 (7.0%)	
Ethnicity	White British	277 (77.6%)	
	Other	80 (22.4%)	
Parents’ marital status	Married/co-habiting	209 (65.1%)	
	Divorced/separated	103 (32.1%)	
	Other	9 (2.8%)	
*Clinical data*			
Diagnoses	AN	145 (40.5%)	
	Atypical AN	145 (40.5%)	
	BN	42 (11.7%)	
	Atypical BN	25 (7.0%)	
%mBMI	AN (n = 145)		77.05 (6.1)
	Atypical AN (n = 145)		88.04 (10.1)
Binge frequency/28 days	BN (n = 35)		13.26 (12.2)
	Atypical BN (n = 19)		7.05 (8.3)
SIV frequency/28 days	BN (n = 34)		23.5 (19.0)
	Atypical BN (n = 20)		9.9 (11.3)
DUED (months)	AN (n = 142)		10.98 (10.2)
	Atypical AN (n = 130)		14.22 (17.0)
	BN (n = 36)		24.60 (16.3)
	Atypical BN (n = 21)		11.9 (8.1)
*Menstrual status*			
AN (n = 135)	Secondary amenorrhea	109 (80.7%)	
	Pre-menarchal	20 (14.8%)	
	Contraceptive pill	6 (4.4%)	
Atypical AN (n = 132)	Periods present	67 (50.8%)	
	Secondary amenorrhea	43 (32.6%)	
	Pre-menarchal	17 (12.9%)	
	Contraceptive pill	5 (3.8%)	
Comorbidity: Self-report measures scored above clinical cut off*			
	MFQ (n = 303)	159 (52.4%)	
	SCARED (n = 304)	170 (55.9%)	
	CHOCI (n = 248)	118 (47.6%)	

*Number of cases varies because of missing data

Abbreviations: *%mBMI* percentage median body mass index, *DUED* duration of untreated illness prior to assessment, *MFQ* mood and feelings questionnaire, *SCARED* screen for child anxiety related disorders, *SD* standard deviation, *SIV* self-induced vomiting

### Psychotherapeutic treatments received within the service

#### AN/Atypical AN

Nearly all the young people (98.6%, n = 286) with a diagnosis of AN/Atypical AN received FT-AN. One hundred and sixty-one of these young people (55.5%) were seen individually for four or more sessions (range 4–59, median = 8). FT-AN was intensified through attendance of up to ten days of multi-family therapy (MFT-AN) for 33.4% (n = 97). The median length of treatment for AN/Atypical AN was 11 months (IQR 10 months). The median number of total number of sessions received was 25 (IQR 23).

#### BN/Atypical BN

Treatment for those with a diagnosis of BN/Atypical BN was more variable with the majority, 86.6% (n = 58), receiving a combination of FT-BN and individual CBT. The remaining 16.4% (n = 11) received just FT-BN and 13.4% (n = 9) individual CBT. In addition, 34.5% (n = 20) attended up to fourteen sessions of Multi-family therapy for BN (MFT-BN), which was initiated in the service during the audit period (2010). The median length of treatment for BN/Atypical BN was seven months (IQR 11 months), which was significantly shorter than for young people with AN/Atypical AN (Mann–Whitney U = 7028, z =  − 3.53, *p* < 0.0001). Median number of family therapy sessions was six (IQR nine) and individual sessions was nine (IQR 12).

### Medication

#### AN/Atypical AN

In the AN/Atypical AN group, 38.3% (n = 111) were prescribed psychotropic medication, with the likelihood of prescription being higher in the group who had enhanced treatment (AIM group) compared to the group who only had outpatient treatment (OPT group) (*χ*^2^ (1, n = 290) = 45.02, *p* < 0.0001; OPT 26.8%, n = 57; AIM 70.1%, n = 54). Of those who were prescribed psychotropic medication, 70.1% (n = 78) were prescribed Olanzapine, and in nearly half of them (n = 38), an antidepressant selective serotonin reuptake inhibitor (SSRI) was added. Olanzapine prescribing was introduced into MCCAED routine clinical practice for AN treatment prior to this service evaluation. MCCAED’s routine psychopharmacologic practice was to start Olanzapine early in treatment (first three months) in cases when the young person displayed high levels of distress and had negligible weight gain trajectory or weight loss. SSRIs were prescribed at later stages of treatment for symptoms of comorbid anxiety or depression.

#### BN/Atypical BN

In the BN/Atypical BN group, 20.9% (N = 14) were prescribed psychotropic medication. Most, (92.9%, n = 13) were prescribed SSRIs for comorbid anxiety and/or depressive disorder.

### Effectiveness of the treatment programme for the whole sample

In AN/Atypical AN group there were significant improvements at the end of treatment in %mBMI (d = 1.03) and EDE-Q Global score (d = 0.9) with a large effect size (see Table 2). There were also significant improvements in the depressive (MFQ), anxiety (SCARED), and obsessive–compulsive symptoms (ChOCI) and quality of life (QoL) on self-reported measures with small and medium effect sizes.

**Table 2:** Changes in clinical measures over treatment for AN/Atypical-AN group

	n (% missing)	Assessment mean (SD)	Discharge mean (SD)	Paired differences mean (SD)	95% CI of difference	t-value	Significance (2 tailed)	Cohen’s *d*
%mBMI	290 (0.0%)	82.55 (9.96)	91.92 (10.18)	9.38 (9.13)	8.32, 10.43	17.49	< 0.001	1.03
EDE-Q Global	190 (34.5%)	3.01 (1.71)	1.45 (1.48)	− 1.55 (1.67)	− 1.87, − 1.23	− 9.70	< 0.001	0.93
QoL	96 (66.9%)	5.74 (2.27)	7.21 (2.06)	1.47 (2.87)	0.72, 0.51	5.01	< 0.001	0.51
MFQ	105 (63.8%)	28.30 (16.58)	16.93 (16.60)	− 11.37 (17.6)	− 14.77, − 7.97	− 6.63	< 0.001	0.65
SCARED	107 (63.1%)	27.82 (17.38)	20.36 (15.46)	− 7.46 (16.55)	− 4.28, − 10.63	− 4.66	< 0.001	0.45
ChOCI	83 (71.4%)	15.35 (11.95)	9.05 (10.69)	− 6.30 (14.49)	− 9.46, − 3.14	− 3.96	< 0.001	0.44

Abbreviations: *ChOCI* Children’s obsessional compulsive inventory, *CI* confidence interval, *EDE-Q* eating disorder examination questionnaire, *MFQ* mood and feelings questionnaire, *QoL* Quality of Life, *SCARED* screen for child anxiety related disorders, *SD* standard deviation

The overall outcome for the AN/Atypical AN group using the Morgan Russell classification is shown in Table 3. Approximately 77% had a good or intermediate outcome at the time of discharge with a significantly greater proportion in the Atypical AN group (*χ*^2^ (2, n = 287) = 6.45, *p* < 0.05).

**Table 3:** Morgan Russell Outcome at end of treatment (AN/Atypical AN)

	All AN/Atypical AN (n = 290)	AN (n = 145)	Atypical AN (n = 145)
Good	169 (58.9%)	74 (51.7%)	95 (66.0%)
Intermediate	51 (17.8%)	28 (19.6%)	23 (16.0%)
Poor	67 (23.3%)	41 (28.7%)	26 (18.0%)
Missing	3	2	1

Abbreviations: *AN* anorexia nervosa

In the BN/Atypical BN group just over half of young people were abstinent from binging and vomiting, with a significantly higher rate of abstinence in the atypical BN group (*χ*^2^ (2, n = 63) = 13.85, *p* < 0.0001) (see Table 4).

**Table 4:** Bulimic symptoms at end of treatment (BN/Atypical BN)

	All BN/Atypical BN (n = 67)	BN (n = 42)	Atypical BN (n = 25)
None	32 (50.8%)	12 (30.8%)	20 (83.3%)
< weekly	5 (7.9%)	5 (12.8%)	0 (0%)
≥ weekly	26 (41.3%)	22 (56.4%)	4 (16.7%)
Missing	4	3	1

Abbreviations: *BN* bulimia nervosa

**Fig. 1:**
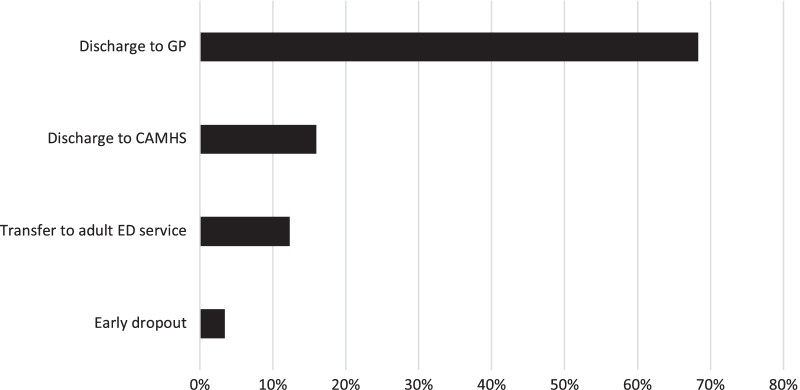
Mode of discharge for all patients. Abbreviations: *CAMHS* child and adolescent mental health service, *ED* eating disorder, *GP* general practitioner

Figure 1 shows the onward referral pathways for young people following their treatment in MCCAED. In most cases these were collaboratively agreed between the treating clinician and the family, although a small number (n = 24; 6.7%) discontinued treatment against clinical advice and were discharged to primary care (GP). Twenty young people who were discharged to primary care with the agreement of the team were classified as having a poor outcome on the Morgan Russell scale because their weight was below 85%mBMI. Fourteen were menstruating and generally doing well despite their low weight and the remainder were maintaining clinical improvements and the family and the therapist agreed to discontinue treatment on the understanding that treatment could restart if this was necessary. Discharge to Child and Adolescent Mental Health Services (CAMHS) was for further treatment of comorbid disorders.

A greater proportion of young people diagnosed with BN/Atypical BN compared to young people with AN/Atypical AN, were discharged to CAMHS (22.1% vs. 13.1%) and less frequently to the GP (52.9% vs. 69.7%), however, this was not statistically significant (*χ*^2^ (3, n = 357) = 7.51, *p* = 0.057). There were significant differences between the OPT and AIM groups in the proportion of young people discharged back to primary care or referred on for further psychiatric care (*χ*^2^ (3, n = 290) = 7.90, *p* = 0.048). Post hoc z test analysis revealed that the AIM group had significantly more referrals to Adult ED services than statistically expected. Of the 357 young people treated and discharged from MCCAED, 18 (5.0%) were re-referred to MCCAED during the study period.

### The receipt of treatment enhancement during outpatient care

Treatment for 26.6% (n = 77) of the young people with AN/Atypical AN was enhanced through day or in-patient treatment. These young people comprise the Additional Intervention and Management (AIM) Group for further analyses. Of these, 53.2% (n = 41) were day patients in ITP, 13.0% (n = 10) had a psychiatric admission and 33.8% (n = 26) had both ITP and psychiatric admissions. The remaining young people were treated solely as outpatients and comprise the Outpatient (OPT, n = 213) group for further analyses.

The treatment of 6.0% (n = 4) of the young people diagnosed with a BN/Atypical BN was enhanced through in-patient psychiatric treatment. These groups were not compared further.

### Duration of treatment for OPT and AIM groups

**Fig. 2:**
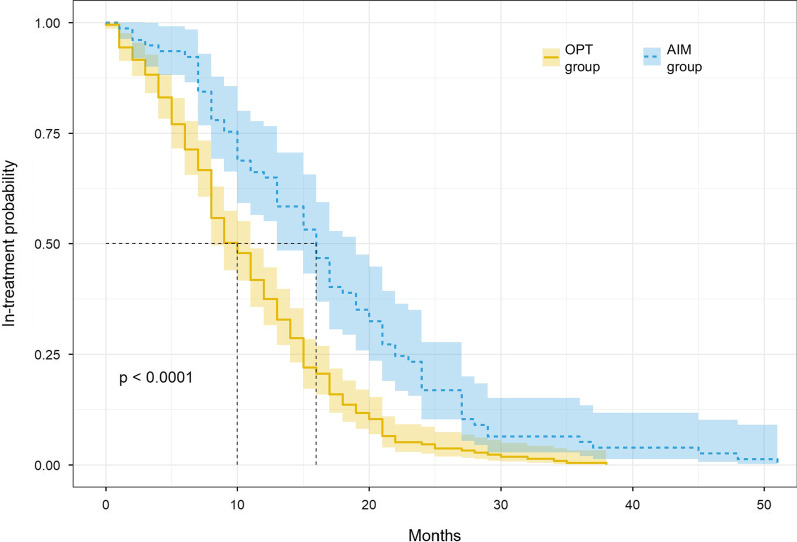
Survival curves according to treatment condition. Abbreviations: *AIM* additional intervention management (day treatment or inpatient treatment, *OPT* outpatient treatment only

Stratified by treatment enhancement or not, median treatment duration for the OPT group was 10 months (95% CI 8, 11) and 16 months (95% CI 13, 19) for the AIM group, with a significant difference by log-rank test (χ^2^ = 25.1, *p* < 0.001). Survival curves stratified by the treatment enhanced group indicator are shown in Fig. 2. After 12 months of treatment, 89/213 (42%) patients were still in treatment in the OPT group versus 51/77 (66%) in the AIM group.

### Factors predictive of treatment enhancement

Descriptive statistics showing difference between groups (OPT vs. AIM) are shown in Table 5. At assessment, the AIM group had significantly higher scores of self-reported ED symptoms (EDE-Q global score), depression (MFQ), and anxiety (SCARED). The AIM group also had significantly lower %mBMI at assessment, and 1 and 3 months into treatment, compared to OPT group.

**Table 5:** Descriptive statistics comparing OPT and AIM group at baseline and early in treatment for AN/Atypical AN patients only

Characteristic	Overall (N = 290)^1^	OPT group (N = 213)^1^	AIM group (N = 77)^1^	*p* Value^2^
Age	15 (13, 16)	15 (13, 16)	15 (13, 16)	0.2
Duration of illness	8 (5, 12)	8 (5, 13)	8 (6, 12)	0.6
Missing	19	14	5	
Age of Onset	14 (12, 15)	14.00 (12, 15)	14 (13, 15)	0.6
Missing	15	11	4	
EDE-Q (Global)	3.3 (1.5, 4.7)	3.1 (1.2, 4.6)	4.1 (2.5, 5.1)	0.002
Missing	44	31	13	
MFQ	28 (18, 43)	25 (14, 40)	36 (23, 50)	< 0.001
Missing	46	34	12	
SCARED	27 (13, 39)	24 (12, 34)	34 (20, 43)	0.002
Missing	45	33	12	
ChOCI	16 (5, 26)	15 (5, 25)	20 (4, 28)	0.3
Missing	92	67	25	
QoL	5 (4, 7)	5 (4, 7)	5 (3, 7)	0.051
Missing	69	50	19	
%mBMI at assessment	81.7 (76.1, 87.4)	82.2 (76.7, 88.6)	79.8 (72.9, 85.0)	0.007
Missing	0	0	0	
%mBMI at 1 month	84.8 (78.2, 91.4)	85.6 (79.9, 93.0)	80.3 (74.5, 86.9)	< 0.001
Missing	3	2	1	
%mBMI at 3 months	87.0 (80.6, 93.5)	87.9 (82.7, 95.3)	82.7 (75.0, 90.3)	< 0.001
Missing	22	18	4	

^1^Median (IQR)

^2^Wilcoxon rank sum test

Abbreviations: *%mBMI* percentage of median body mass index, *AIM* additional intervention management (day treatment or inpatient treatment; *ChOCI* Children’s obsessional compulsive, *CI* confidence interval, *DUED* duration of untreated illness prior to assessment, *EDE-Q (G)* eating disorder examination questionnaire global score, *MFQ* mood and feelings questionnaire, *OPT* outpatient treatment only, *QoL* Quality of Life, *SCARED* screen for child anxiety related disorder, *SD* standard deviation

**Fig. 3:**
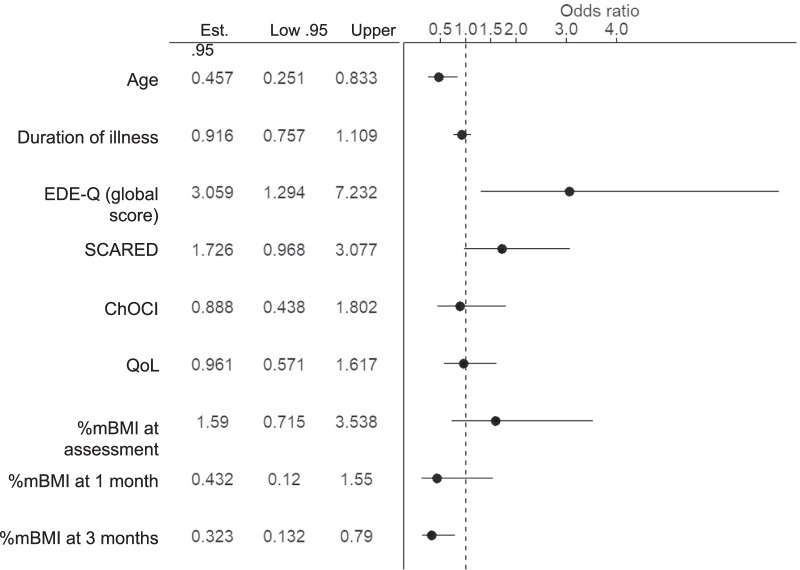
Estimated odds ratios and 95% confidence intervals for need for additional treatment (AIM group) versus no additional treatment (OPT group) according to baseline variables. Abbreviations: *%mBMI* percentage of median body mass index, *ChOCI* Children’s obsessional compulsive inventory, *EDE-Q* eating disorder examination questionnaire, *QoL* Quality of Life, *SCARED* screen for child anxiety related disorders

The adjusted odds ratios showing the associations between baseline variables, including %mBMI at assessment, 1 month and 3 months into treatment, and receipt of enhanced treatment are shown in Fig. 3. By far the strongest predictor (over 60% of chi-squared) of whether enhanced treatment was added, was %mBMI at month 3 of treatment (OR 0.27; 95% CI 0.16, 0.45). More modestly important predictors (circa 15% of chi-squared each) were EDE-Q scores (OR 2.27; 95% CI 1.13, 4.59) at assessment and age (0.59; 95% CI 0.25, 0.83). Older patients had lower odds of enhanced treatment. Patients with higher EDE-Q scores at assessment had greater odds of enhanced treatment. The rest of the predictors were not important and had OR relatively close to 1.

### Comparison of outcomes for AIM and OPT groups

Global outcomes on the Morgan Russell Criteria at discharge are presented in Table 6. There were significant differences between the OPT and AIM groups in the proportion of young people categorised as having a good, intermediate or poor outcome on the Morgan Russel criteria (*χ*^2^ (2, n = 287) = 6.59, *p* < 0.05). Post hoc z test analysis revealed that the AIM group had significantly fewer good outcomes and significantly more poor outcomes than statistically expected.

**Table 6:** Outcome on Morgan-Russell criteria (AN/Atypical AN)

	OPT group (n = 213)	AIM group (n = 77)
Good	133 (63.0.%)	36 (47.4%)
Intermediate	36 (17.1%)	15 (19.7%)
Poor	42 (19.9%)	25 (32.9%)
Missing	2	1

Abbreviations: *AIM* additional intensive management (day treatment or inpatient treatment), *OPT* outpatient treatment only

**Fig. 4:**
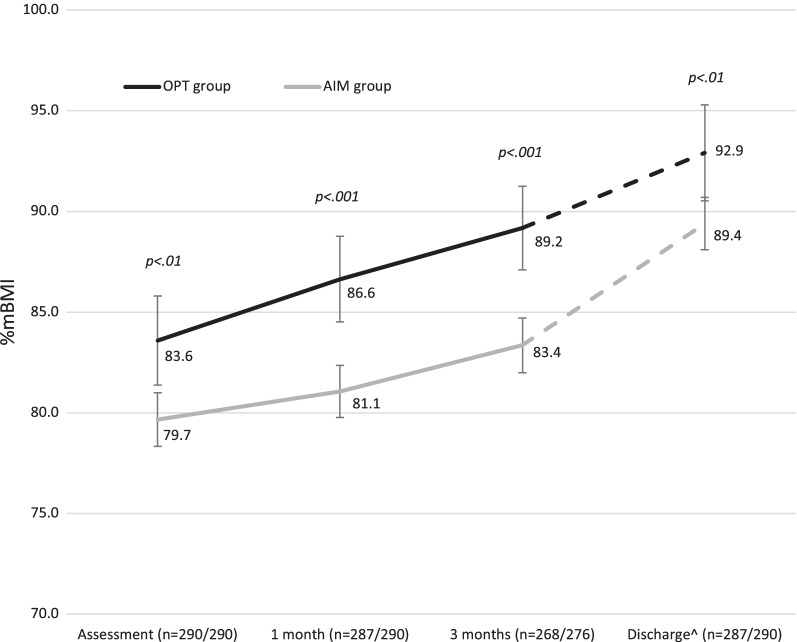
Mean weights and 95% confidence intervals for OPT and AIM groups. ^Dotted line used to emphasise that time to discharge is variable for each patient. Abbreviations: *AIM* additional intensive management (day treatment or inpatient treatment), *OPT* outpatient treatment only

Weight trajectories during treatment for each group are presented in Fig. 4. However, at discharge there were no significant differences between the AIM and OPT group on self-reported ED symptoms (EDE-Q global score), anxiety (SCARED), depression (MFQ), OCD symptoms (ChOCI) and quality of life (QoL) (all *p*’s > 0.05). See Additional file 1: Additional statistical analyses.

As discussed in the methods section, to assess equivalence for discharge outcome between the AIM and OPT groups, Morgan Russell outcomes were fitted into ordinal regression model adjusted for %mBMI at assessment, 1 and 3 months of treatment. There was a small difference between the AIM and OPT groups at discharge in terms of the Morgan Russell criteria. The point estimate of the odds of a poorer outcome in the AIM treatment group were 1.26 relative to the OPT group, however, the 95% CI ranged from 0.71 to 2.25 compatible with no effect of treatment group (*p* < 0.42). Similarly, when %mBMI was adjusted for pre-treatment assignation, %mBMI did not differ between groups, mean difference =  − 0.38 (95% CI − 2.44, 1.68, *p* = 0.72).

## Discussion

This study aimed to evaluate the effectiveness of empirically supported treatments in a real-word, multidisciplinary, specialist ED service setting.

### Clinical and demographic characteristics of the young people referred to the service

All consecutive referrals over a period of five years to MCCAED, the primary provider of specialist treatments for children and adolescents with EDs for a population of approximately 2.2 million people, were included in this study. MCCAED provides a number of empirically supported treatments (FT-AN, FT-BN, MFT-AN, MFT-BN). However, in MCCAED’s routine clinical practice, treatments are offered flexibly, and different treatment modalities may be combined, if clinically appropriate. For example, family therapy can be combined with individual therapy or individual, family and multifamily therapy can be combined together for both AN/Atypical AN and BN/Atypical BN presentations. Similarly, duration of treatment can vary depending on the specific needs of each individual and their family.

The great majority of young people referred to MCCAED were females with food restriction as their main symptom. They were equally diagnosed with AN and Atypical AN. Less than one fifth of the whole sample were diagnosed with BN or Atypical BN. The number of young people with BN appears disproportionally low when compared to expected prevalence in the community. In the National Comorbidity Survey in USA of youth aged 13–18 years [36] the lifetime prevalence rate of BN was 0.9% and 12-month prevalence 0.6% indicating that overall prevalence of BN in adolescence in a community sample is not very different to the prevalence of AN. There might be a number of reasons why young people with BN are not seeking or delaying accessing treatment, including stigma, secretiveness and shame associated with the disorder.

There are also likely social and cultural factors impacting treatment seeking, as has been consistently reported in the literature [37–39]. It has been demonstrated that ethnic minority communities in the UK and USA are less likely to seek and receive treatment, be diagnosed with an ED or referred to specialist treatment [38]. This discrepancy is mirrored in the current study, in which, the majority self-identified as White British (78%), however, the 2011 census, completed around the time of the current audit, identified that inner London had a white population of approximately 60% [40].

### Effectiveness of the treatments

Young people with BN/Atypical BN had a significantly shorter length of treatment compared to young people with AN/Atypical AN and their treatment more often combined individual treatment and family therapy (86.6% vs. 55.5%). Just over half (50.8%) of the young people with BN/Atypical BN were abstinent from binging and purging at the end of treatment in MCCAED and their outcomes compared favourably to RCTs of BN treatment that recruited young people with BN and partial BN [41–43]. In RCTs, abstinence from binge-purge behaviours range between 12–39% at the end of six months of treatment and 29–44% at six-month follow-up [41–43].

In the current study young people with BN/Atypical BN reported high levels of anxiety and significantly more severe depression symptoms compared to young people with AN/Atypical AN. It has been well established that comorbidity is common in BN. High levels of psychiatric comorbidity have been reported in a treatment seeking sample of adolescents [44] and up to two thirds of adults with BN have a comorbid lifetime anxiety disorder, frequently with onset in childhood [45]. The high level of comorbidity in our sample likely contributed to over 20% of the young people with BN/Atypical BN getting discharged at end of treatment with MCCAED to CAMHS, for further treatment of these comorbid disorders.

For young people with AN/Atypical AN, the median length of treatment (11 months) was similar to the 12-month length of treatment described in some RCTs [46, 47]. As this was treatment in the real word, the length of treatment was variable. The median total number of sessions (N = 25) was higher than the maximum number of sessions (N = 16–24) offered in RCTs [47–49]. The higher number of the total session is possibly partially influenced by MCCAED’s more flexible approach of offering a combination of individual and family therapy sessions to most young people.

It has been shown that in FBT, parent alliance with the therapist is often stronger than that of the young people [50]. In a recent Swedish cohort study [17], of young people with AN who predominantly received outpatient treatment (93%), participants were asked to evaluate different aspects of their relationship with their therapist. They highly rated the therapist’s ability to listen and therapist’s knowledge about ED, but rated lower the therapist’s ability to help them, their own participation in treatment and their agreement with the therapist about how treatment should be conducted. Young people felt that some of the treatment goals they considered important (e.g., how to handle strong emotions like sadness and anxiety or learning how to eat normally) were not fulfilled in therapy. Young people who received individual therapy were generally more satisfied with their therapy than those receiving any other form of therapy. Ensuring young people are involved in family treatment and offering some individual time might be beneficial for developing a good working alliance, trust and fulfilling their personal goals in treatment. Combining individual sessions and family sessions might have contributed to the relatively low early dropout rate (3%) and disengagement from treatment (6.7%) for the whole sample in this study.

At the end of treatment young people with AN/Atypical AN improved greatly in regard to their weight and eating disordered symptomatology and moderately in relation to anxiety, depressive, obsessive–compulsive symptoms and quality of life. The number of young people under the age 18, re-referred to MCCAED during the four-and half year study period, was low (5%).

Their overall outcomes were classified using the Morgan Russell criteria, as this was a classification previously used in the service. The main treatment model used in the service, FT-AN [21], does not set a universal weight goal such as 95%mBMI or 100%mBMI as a treatment target. Rather, healthy weight is individually determined for each person. Defining remission as achieving a general weight target such as 95%m BMI would be inconsistent with the service model that 'one size does not fit all’. FT-AN aims to support the young person to achieve physical health first (e.g., regaining periods, not having signs of starvation or malnutrition, understanding current weight in the context of the individual’s weight history) and recognises that psychological and cognitive recovery often lags behind weight restoration. The lack of evidence-based methods or consensus on how to calculate expected target weight in treatment [51] and the lack of consensus in defining outcome and recovery [52] has been identified in the literature. Discussions within the ED field are moving towards recognising that defining outcome as remission or recovery on the basis of solely physical aspects without considering psychological, social or cognitive functioning is not sufficient.

The decision to use the Morgan-Russell global outcome served the sole purpose of classifying physical improvements and allowing for comparison with previous trials that have used the same outcome measure. A small number of young people in our study were classified as having poor outcome on Morgan-Russell scale because of their weight, however, they were menstruating and discharge from the service was collaboratively agreed between family and therapist. This highlights the difference between defining outcome for clinical as opposed to research purposes. In a research setting outcomes need to be clearly specified and predefined and are generally assessed at a predetermined point in time. Individual variability means that there will inevitably be a proportion of false positives and false negatives (and changing the outcome criteria will often just shift the balance between type I and type II errors). In the clinical context the focus is on individual functioning and progress is assessed by drawing on broader, individual specific information, and change over the course of treatment.

Overall, young people with AN/Atypical AN in our study had very similar percentage of the combined good and intermediate outcomes on the Morgan-Russell classification at discharge compared to subjects at the end of a 12-month multi-centre RCT trial of FT-AN and MFT-AN for adolescent anorexia nervosa (76.8% vs. 75%) (46). In this RCT, young people who had MFT-AN in addition to FT-AN had superior outcome compared to young people who had FT-AN alone. At face value, the percentage of young people having a good outcome at discharge from our service is higher than in the RCT (57.2% vs. 45%). However, the higher %mBMI at assessment (82.5% vs. 78%) and varied length of treatment in our service evaluation could explain the observed difference. Weight at assessment has been consistently shown to be a predictor of treatment outcome indicating that subjects with a lower %mBMI at assessment are less likely to achieve remission when this is defined as 95%mBMI at 12-month follow-up [12, 53–55]. A higher BMI at assessment has also been shown to predict better outcomes at the end of treatment and follow-up in a recent systematic review [56].

Around a quarter of patients with AN/Atypical AN were prescribed olanzapine, and significantly more often in young people who were admitted to the day programme/inpatient treatment. In another retrospective patients record review study in a Canadian tertiary treatment centre, 14% of patients were prescribed Olanzapine [57]. Though conclusive evidence is lacking regarding the benefits of Olanzapine in treatment of adolescent AN, in a recent survey of child and adolescent ED psychiatrists in England on their medication prescribing practices in the treatment of AN, Olanzapine was reported as the most commonly prescribed medication for AN by 38% of the respondents [58]. The use of SSRIs in our study was solely for treatment of anxiety and depressive disorders once weight gain had been established.

### The receipt of treatment enhancement during outpatient care

Secondary aims of this study were to evaluate the extent to which routine outpatient treatment required additional day- and in-patient care and to analyse the effectiveness of combined outpatient and day/in-patient treatment. More than a quarter of young people in our study were admitted to the day program (ITP) and/or inpatient treatment (AIM group). In RCTs that have tested FBT, MFT-AN, FT-AN, systemic family therapy or Adolescent Focused Therapy, depending on the treatment arm and health setting, hospitalization rates range from 6 to 32% [46, 47, 59]. The significantly longer treatment in the AIM group (16 months) is similar to the average treatment duration of approximately 15 months found in another naturalistic study that included inpatient, day patient and/or outpatient treatment [16].

### Factors predictive of treatment enhancement

In this study, %mBMI at 3 months of treatment was the strongest predictor of the receipt of treatment enhancement. Eating disorder symptom severity (EDE-Q global score) and age at assessment also predicted this, albeit less strongly. This is consistent with findings that young people who show rapid weight gain at the start of treatment are more likely to remit or have favourable outcome at the end of one-year treatment [60, 61].

### Comparison of treatments for AIM and OPT groups

In the AIM group 45.5% had good, and 32.5% had poor Morgan-Russell global outcome. In the RCT [62] that compared inpatient and day patient treatment after medical stabilisation one year after patients were admitted to hospital, only 25–31% had a good outcome, and 56–60% had poor outcome on Morgan-Russell global outcome, suggesting poorer global outcomes in the RCT than in the current study.

There were no significant differences in ED, depressive, anxiety and obsessive–compulsive symptoms, or quality of life at the end of treatment between the AIM and OPT group. There were significant differences on Morgan-Russell outcomes (fewer good, more poor outcomes) and %mBMI (lower for AIM group). However, when analyses of equivalence for outcomes at discharge between the AIM and OPT groups were adjusted for %mBMI at assessment, 1 and 3 months into treatment, there were only small differences and confidence intervals indicated no effect of treatment group. Similarly, %mBMI was not significantly different between the groups.

The majority of young people in the AIM group had a combination of FT-AN, individual therapy and FT-AN based day programme treatment. It appears that with the combination of family/individual/day programme treatment, and longer duration of treatment, most of the young people who would have been predicted to have a less favourable outcome at the start of treatment were able to catch up with the recovery process by the end of treatment. Their outcomes seem in many respects to be similar to outcomes of the young people who started treatment at a higher %mBMI, and received only outpatient treatment, both usually predictive of a good outcome.

## Limitations

There are several important limitations to this study. Most notable is the variable amount of missing data across the different measures. Missing self-report data at discharge could also suggest a selection bias in outcome measures favouring those who experienced improvements, which needs to be considered when interpreting the results. MCCAED also has a flexible approach to treatment planning and delivery using additional interventions (e.g., enhanced treatments, combination of individual and family therapy, olanzapine), whereas treatment evaluation in RCTs typically follow a manualized protocol. Hence, comparing the performance of specific, empirically supported interventions in specialist clinical settings versus research setting is also very tentative.

## Conclusions

This study supports emerging evidence that empirically supported treatments perform equally well in an everyday specialist clinical setting as in research settings. Outcomes for young people with AN/Atypical AN and BN/Atypical BN were equivalent or better than RCT outcomes. Treatment for a quarter of young people treated in our community specialist ED service was enhanced, mostly with a day programme or a combination of day programme and inpatient admissions. Young people whose treatment was enhanced had a longer duration of treatment compared to young people who only had outpatient treatment. This study shows that enhancing treatment with day programme and/or inpatient admissions is associated with favourable outcome for most of the young people in our study.

## Supplementary Information

**Additional file 1:** Additional statistical analyses.

## Data Availability

Data are available from the corresponding author on reasonable request.

## Abbreviations

%mBMI: Percentage median body mass index
AIM: Additional intervention and management group
ANOVA: Analysis of variance
ARFID: Avoidant restrictive food intake disorder
BN: Bulimia nervosa
CBT: Cognitive behavioural therapy
CBT-E: Cognitive behaviour therapy for eating disorders
CAMHS: Child and adolescent mental health service
ChOCI: Children’s obsessional compulsive inventory
CI: Confidence interval
DUED: Duration of untreated eating disorder
ED: Eating disorder
EDE-Q: Eating disorder examination questionnaire
EDNOS-R: Eating disorder not otherwise specified predominantly characterised by restriction
EDQLS: Eating disorder quality of life scale
FT-AN: Family therapy for anorexia nervosa
FT-BN: Family therapy for bulimia nervosa
GP: General practitioner
ICD 10: International classification of diseases (10th edition)
IQR: Interquartile range
ITP: Intensive treatment programme
MCCAED: Maudsley centre for child and adolescent eating disorders
Mdn.: Median
MFT-AN: Multi-family therapy for anorexia nervosa
MFT-BN: Multi-family therapy for bulimia nervosa
MFQ: Mood and feelings questionnaire
NHS: National health system
OPT: Outpatient treatment group
QoL: Quality of life
RCT: Randomized controlled trial
SCARED: Screen for child anxiety related emotional disorders
SD: Standard deviation
SIV: Self-induced vomiting
SPSS: Statistical package for social sciences
SSRI: Selective serotonin reuptake inhibitor
UK: United Kingdom
USA: United States of America

## Footnotes

1 Treatments listed were provided at the time of the study. Since the study completion additional treatments have been added in the routine clinical practice: Radically Open Dialectical Behaviour Therapy, Attachment Based Family Therapy, Cognitive Behavioural Therapy for Eating Disorders (CBT-E). Several of these have also been added to ITP [19]. Since 2019 MCCAED includes a service for children and adolescents with Avoidant Restrictive Food Intake Disorder (ARFID).
