# Supplementary material for: From efficacy to effectiveness: child and adolescent eating disorder treatments in the real world (part 1)—treatment course and outcomes
Source: J Eat Disord. 2022 Feb 21;10:27. doi: 10.1186/s40337-022-00553-6 (PMC8862310; doi:10.1186/s40337-022-00553-6)
Supplement: Supplementary file 1 — Additional file 1: Additional statistical analyses. [file 40337_2022_553_MOESM1_ESM.pdf]

Supplementary Materials | Statistical analyses not reported in main text

Further details of Analysis 4:

The performance of the model was assessed with estimates of discrimination (how well the model distinguished between those who needed additional treatment or not) and calibration or goodness of fit (the correspondence between predicted probabilities and observed data) Discrimination was assessed with the concordance statistic (C-statistic) and R^2. Calibration was assessed graphically with the slope and intercept of a calibration plot of predicted versus actual probabilities. A well calibrated model will have a calibration plot with an intercept of 0 and slope of 1. Both discrimination and calibration were corrected for overfitting or optimism by bootstrap resampling (300 samples). Supplementary material for supporting analyses not reported in the main text.

Model performance for predictors analysis of treatment enhancement:

Internal validation of the performance of the model corrected for optimism is shown in Table S1 Optimism was not insignificant with an R2 correction of 7% (or 25% of the uncorrected. Calibration was relatively weak with a negative intercept indicating global over-estimation of the risk of additional treatment and a slope of 0.82 consistent with overestimation of low risk and underestimate of high risk (Figure S).

Table S1.1 | Model performance statistics for logistic model for Added Treatment or not.

	Original Index	Optimism	Corrected Index
C-Index	0.78	0.04	0.74
R2	0.31	0.07	0.24
Intercept	0.00	0.15	−0.15
Slope	1.00	0.18	0.82

Figure S1.2 | Calibration curve for model predicting additional treatment

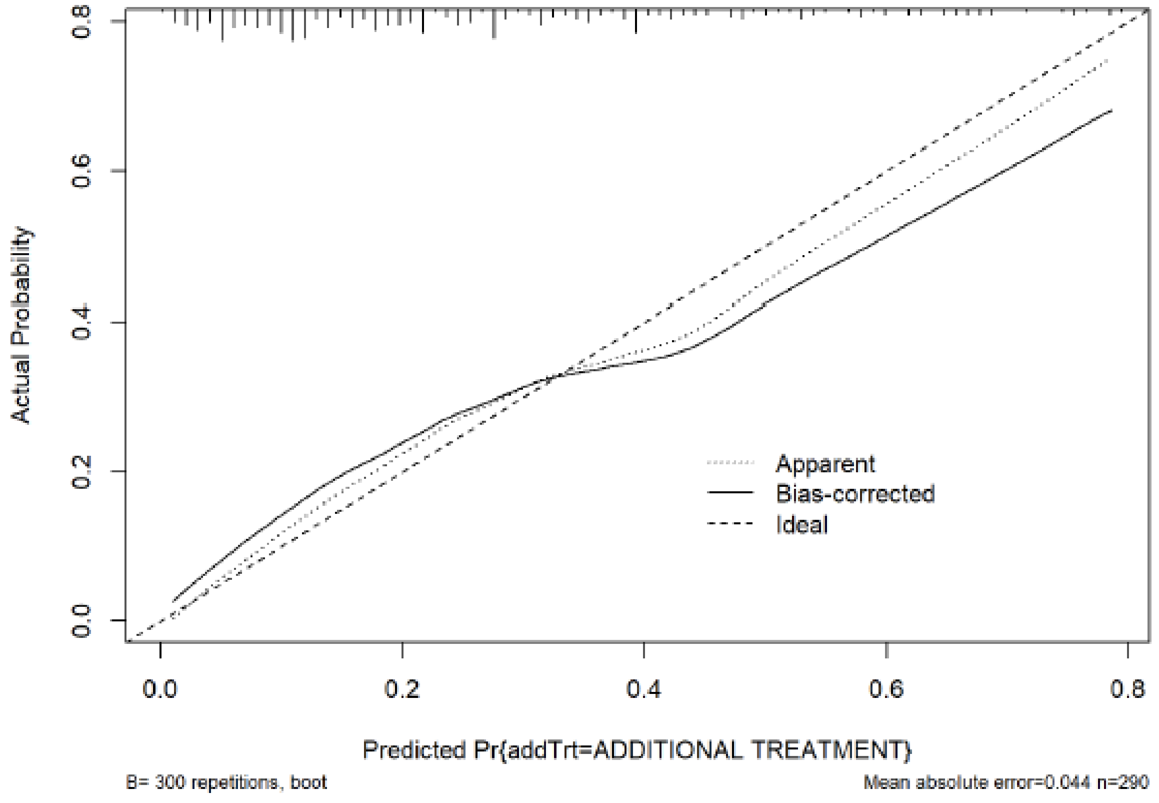


```
##
## n=290   Mean absolute error=0.044   Mean squared error=0.00246
## 0.9 Quantile of absolute error=0.082
```

Predictors of additional treatment

Figure S1.3 | Variable importance by proportion of total model Chi-squared

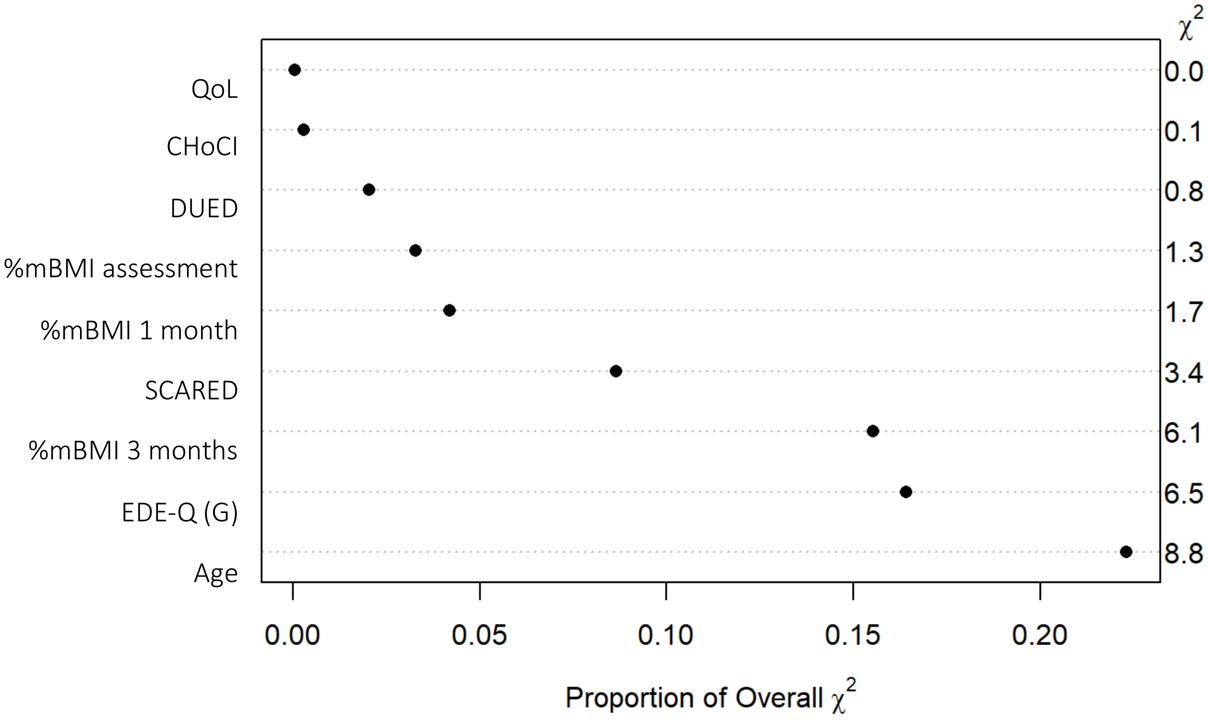

Variable importance determined by Wald statistics, specifically by the proportion of model chi-squared contributed by each predictor to the total model chi-squared (compared to a model with the intercept only). Variables with a larger chi-squared (minus the degrees of freedom) will be more important.

%mBMI Trajectories

Figure S1.4 | Plotting weight trajectories of patients

AN/Atypical AN Patients

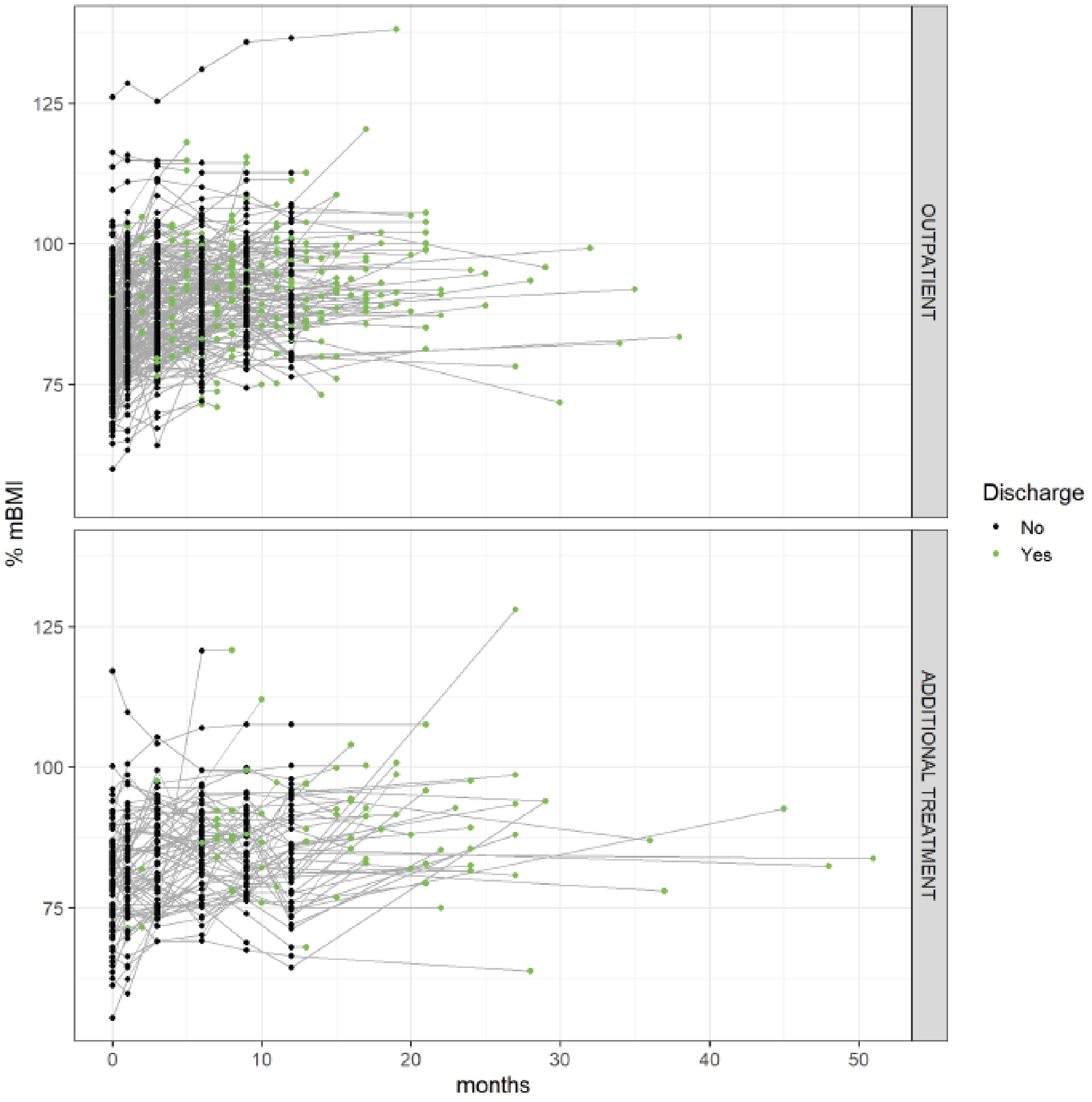

Figure S1.5 | Prediction log odds of additional treatment according to age and %mBMI

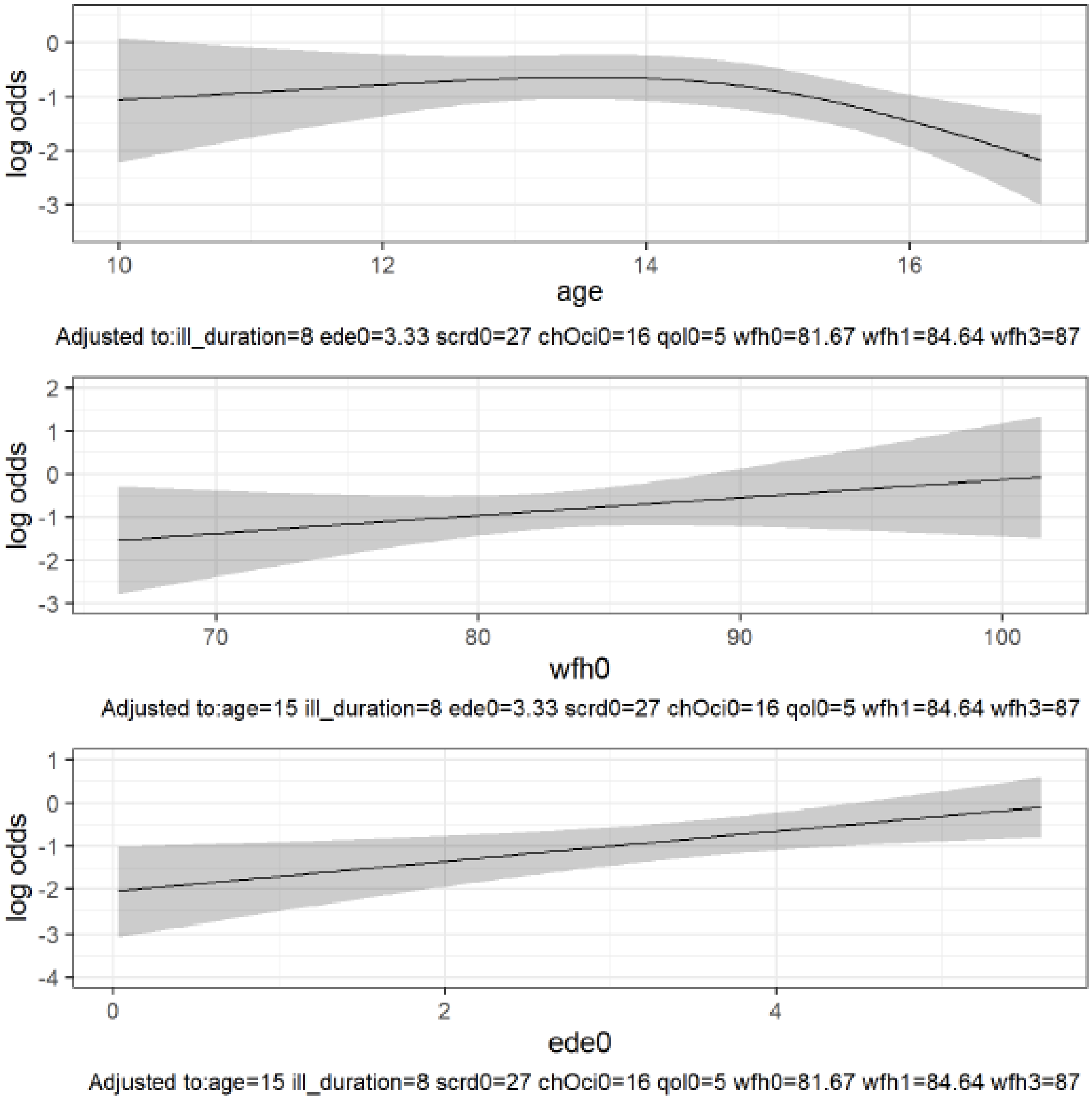

Survival analysis

Figure S2.1 | Predicted for %mBMI and Age

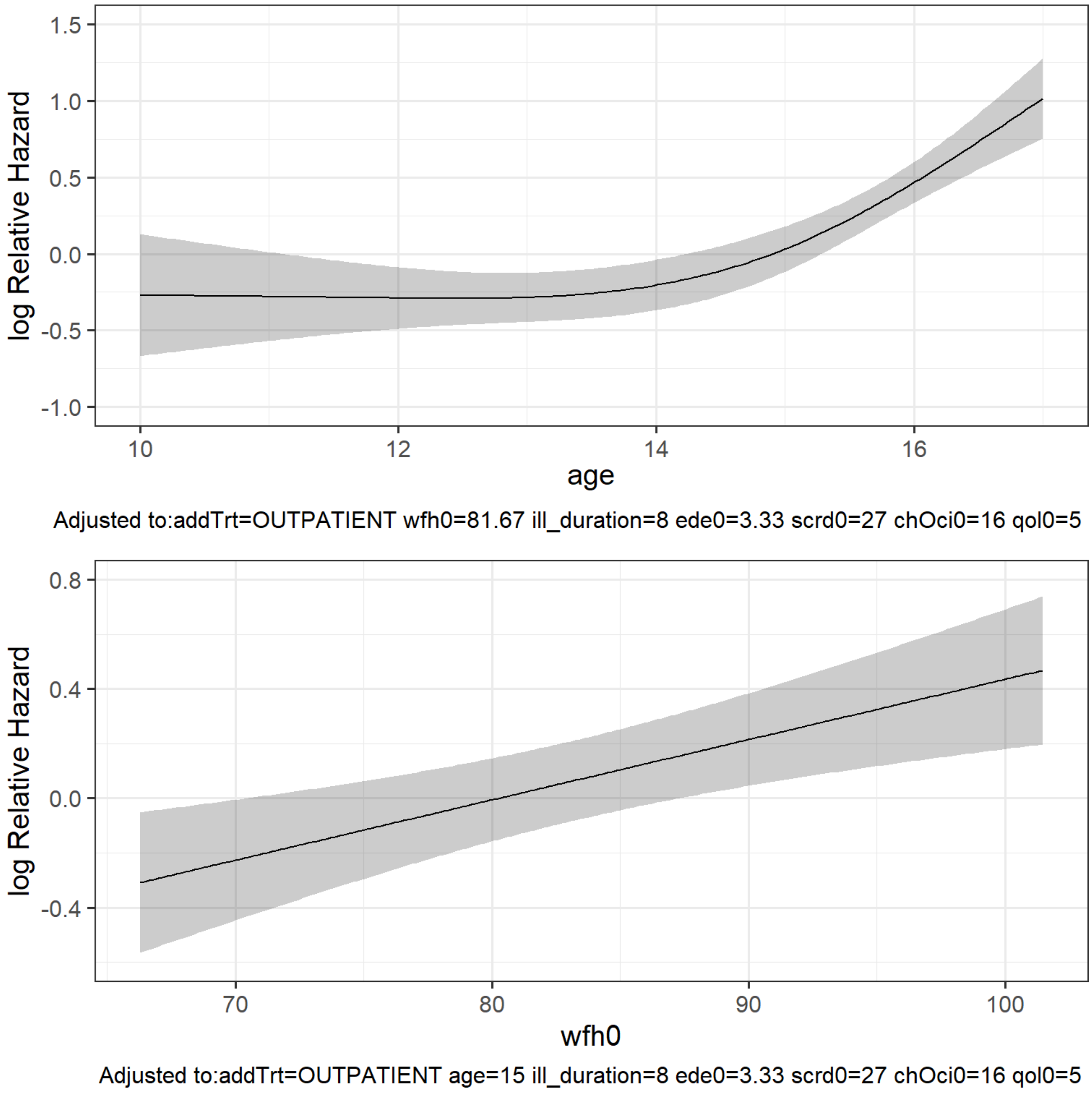

OPT / AIM comparison

Table S3.1 | Descriptive statistics comparing OPT and AIM group at discharge for AN / Atypical AN patients only

	**OPT group** (N = 213)[1]	**AIM group** (N = 77)[1]	**Mean difference 95% CI**	**p-value**[2]
EDE-Q (Global)	1.35 (1.43)	1.60 (1.61)	-0.25	0.44
Missing	121	46	-0.91, 0.32	
MFQ	15.47 (14.07)	20.45 (19.52)	-4.98	0.20
Missing	123	46	-12.67, 2.7	
SCARED	18.54 (14.00)	25.29 (20.00)	-6.75	0.09
Missing	124	46	-14.59, 1.09	
ChOCI	3.68 (5.52)	4.61 (6.29)	-0.93	0.47
Missing	131	46	-3.51, 1.65	
QoL	7.26 (2.05)	7.50 (2.19)	-0.24	0.61
Missing	127	49	-1.19, 0.7	

[1] Mean (SD)

[2] Independent samples t test
